# Supplementary material for: An open-source framework for large-scale, flexible evaluation of biomedical text mining systems
Source: J Biomed Discov Collab. 2008 Jan 29;3:1. doi: 10.1186/1747-5333-3-1 (PMC2276192; doi:10.1186/1747-5333-3-1)
Supplement: Additional file 1 — Evaluation platform source code. The additional file contains the software evaluation framework discussed in this paper. The most current release can be downloaded from SourceForge.net [1]. See the accompanying README file for instructions on installation and use. Also included in the distribution is a collection of UIMA wrappers for some commonly used BioNLP tools and annotated corpora. [file 1747-5333-3-1-S1.zip › bionlp_uima_repository-1.0.1/docs/api/edu/uchsc/ccp/util/nlp/mention/ComplexSlotMention.html]

ComplexSlotMention (Center for Computational Pharmacology Util/UIMA API)


|  |  |  |
| --- | --- | --- |
| **Overview** \| **Package** \| **Class** \| **Tree** \| **Deprecated** \| **Index** \| **Help** | |  |
| **PREV CLASS**   **NEXT CLASS** | **FRAMES**    **NO FRAMES**     **All Classes** |  |
| SUMMARY: NESTED \| FIELD \| CONSTR \| METHOD | DETAIL: FIELD \| CONSTR \| METHOD |  |


---


## edu.uchsc.ccp.util.nlp.mention Class ComplexSlotMention

```
java.lang.Object
  edu.uchsc.ccp.util.nlp.mention.Mention
      edu.uchsc.ccp.util.nlp.mention.ComplexSlotMention
```

**All Implemented Interfaces:**: Comparable

---

``` public class ComplexSlotMention extends Mention ```

---

**Constructor Summary**

`ComplexSlotMention()`
  
          **Deprecated.**

`ComplexSlotMention(String mentionName)`


| **Method Summary** | |
| --- | --- |
| `void` | `addClassMention(ClassMention cm)`             Add a class mention as a slot filler for this complex slot |
| `int` | `compareTo(Object o)`             Must be implemented by subclasses of Mention |
| `boolean` | `equals(Object obj)` |
| `List<ClassMention>` | `getClassMentions()`             Get the class mentions that fill this complex slot |
| `String` | `getDocumentLevelSingleLineRepresentation()` |
| `String` | `getHashKey()` |
| `String` | `getSingleLineRepresentation()`             Return a string representation of this ComplexSlotMention in a single line.  Representation: " [mentionName]: [single-line-rep of ta linked to class mention]" |
| `int` | `hashCode()` |
| `static void` | `printMention(ComplexSlotMention csm, PrintStream ps, int indentLevel)` |
| `void` | `setClassMentions(List<ClassMention> classMentions)`             Set the class mentions that fill this complex slot |

**Methods inherited from class edu.uchsc.ccp.util.nlp.mention.Mention**

`getMentionName, setMentionName`

**Methods inherited from class java.lang.Object**

`clone, finalize, getClass, notify, notifyAll, toString, wait, wait, wait`

**Constructor Detail**

### ComplexSlotMention

```
@Deprecated
public ComplexSlotMention()
```

:   **Deprecated.**

---


### ComplexSlotMention

```
public ComplexSlotMention(String mentionName)
```


**Method Detail**

### getClassMentions

```
public List<ClassMention> getClassMentions()
```

:   Get the class mentions that fill this complex slot

    :   **Returns:**

---


### setClassMentions

```
public void setClassMentions(List<ClassMention> classMentions)
```

:   Set the class mentions that fill this complex slot

    :   **Parameters:**: `classMentions` -

---


### addClassMention

```
public void addClassMention(ClassMention cm)
```

:   Add a class mention as a slot filler for this complex slot

    :   **Parameters:**: `cm` -

---


### getSingleLineRepresentation

```
public String getSingleLineRepresentation()
```

:   Return a string representation of this ComplexSlotMention in a single line.  
    Representation: " [mentionName]: [single-line-rep of ta linked to class mention]"

    :   **Overrides:**: `getSingleLineRepresentation` in class `Mention`
    :   **Returns:**

---


### getDocumentLevelSingleLineRepresentation

```
public String getDocumentLevelSingleLineRepresentation()
```

:   **Overrides:**: `getDocumentLevelSingleLineRepresentation` in class `Mention`

---


### printMention

```
public static void printMention(ComplexSlotMention csm,
                                PrintStream ps,
                                int indentLevel)
```

---


### getHashKey

```
public String getHashKey()
```

---


### compareTo

```
public int compareTo(Object o)
```

:   **Description copied from class: `Mention`**
:   Must be implemented by subclasses of Mention

    :   **Specified by:**: `compareTo` in interface `Comparable` **Overrides:**: `compareTo` in class `Mention`

---


### equals

```
public boolean equals(Object obj)
```

:   **Overrides:**: `equals` in class `Object`

---


### hashCode

```
public int hashCode()
```

:   **Overrides:**: `hashCode` in class `Object`


---


|  |  |  |
| --- | --- | --- |
| **Overview** \| **Package** \| **Class** \| **Tree** \| **Deprecated** \| **Index** \| **Help** | |  |
| **PREV CLASS**   **NEXT CLASS** | **FRAMES**    **NO FRAMES**     **All Classes** |  |
| SUMMARY: NESTED \| FIELD \| CONSTR \| METHOD | DETAIL: FIELD \| CONSTR \| METHOD |  |


---
